# Droplet-based microfluidic screening and sorting of microalgal populations for strain engineering applications^☆^

**DOI:** 10.1016/j.algal.2021.102293

**Published:** 2021-06

**Authors:** Ziyi Yu, Katrin Geisler, Tina Leontidou, Rosanna E.B. Young, Sofie E. Vonlanthen, Saul Purton, Chris Abell, Alison G. Smith

**Affiliations:** a Department of Chemistry, University of Cambridge, Lensfield Road, Cambridge CB2 1EW, UK; b Department of Plant Sciences, University of Cambridge, Downing Street, Cambridge CB2 3EA, UK; c Institute of Structural & Molecular Biology, University College London, Gower Street, London WC1E 6BT, UK

**Keywords:** Microalgae, Fluorescence detection, Microfluidics, Microdroplets, Screening for transformants

## Abstract

The application of microfluidic technologies to microalgal research is particularly appealing since these approaches allow the precise control of the extracellular environment and offer a high-throughput approach to studying dynamic cellular processes. To expand the portfolio of applications, here we present a droplet-based microfluidic method for analysis and screening of *Phaeodactylum tricornutum* and *Nannochloropsis gaditana*, which can be integrated into a genetic transformation workflow. Following encapsulation of single cells in picolitre-sized droplets, fluorescence signals arising from each cell can be used to assess its phenotypic state. In this work, the chlorophyll fluorescence intensity of each cell was quantified and used to identify populations of *P. tricornutum* cells grown in different light conditions. Further, individual *P. tricornutum* or *N. gaditana* cells engineered to express green fluorescent protein were distinguished and sorted from wild-type cells. This has been exploited as a rapid screen for transformed cells within a population, bypassing a major bottleneck in algal transformation workflows and offering an alternative strategy for the identification of genetically modified strains.

## Highlights

• Droplet-based microfluidic systems are promising tools for algal single cell analysis.
• Improved intracellular fluorescence detection allows effective sorting of algae cells.
• The physiological status of single encapsulated algae cells can be determined.
• Sorting in microdroplets enables faster identification of transformed cells.

## 1 Introduction

Considerable attention has been directed recently to the exploitation of microalgae as a sustainable source for autotrophic bioproduction of food, fuels, and especially high value products such as long-chain polyunsaturated fatty acids, terpenoids, pigments, and therapeutic proteins [[1], [2], [3], [4]]. The cultivation of microalgae does not compete with land crops for resources and can be achieved on non-arable land [5]. Some algal species have very high photosynthetic growth rates and under the right conditions are capable of accumulating extremely high levels of metabolites of interest. The last decade has witnessed dramatic progress in microalgal genetic engineering to improve the production of valuable compounds, nonetheless the isolation and analysis of high-performing strains is still labour-intensive and time-consuming. For example, whilst basic tools for genetic engineering such as selectable markers, reporters, and promoter elements are available [[6], [7], [8]], the identification of genetically-modified strains is a slow process. The recovery of transformed lines can take several weeks as colonies need to grow to a sufficient size on selective plates. Furthermore, since integration of the transgene typically occurs at random loci in the nuclear genome, the level and stability of transgene expression can vary significantly amongst transformant lines [9]. Consequently, many lines need to be analysed for the stable expression of the transgene and evaluated for the desired phenotypic change.

A common and sensitive method to monitor microalgal growth is *via* chlorophyll fluorescence, but other fluorescent markers can also be used with these organisms. For example, fluorescent dyes such as Nile Red or BODIPY stain the neutral lipids of microalgae and can be a fast method to quantify the lipid content, avoiding time-consuming and costly gravimetric analysis [10]. Similarly, labelling algal cells with fluorescent proteins has been used in a range of species to study gene expression or *in vivo* protein localisation [[11], [12], [13], [14], [15]]. To date, these intracellular fluorescence measurements have mainly focused on the bulk populations of algal cells in solution or as colonies on solid media. However, the approach also offers the means to understand molecular processes at the single-cell level, by coupling single-cell handling with high throughput fluorescence analysis. This allows stochastic variations within a population of cells to be identified and characterised.

In recent years, droplet-based microfluidic systems have emerged that allow the study of hundreds to a few thousands of individual cells under precisely-defined conditions [[16], [17], [18], [19], [20]]. Each pico- or nano-litre volume microdroplet serves as an isolated microreactor, enabling numerous parallel chemical and biological analyses under various cell culture conditions. Several studies have shown that microdroplets allow the isolation and cultivation of individual microalgal cells, enabling the identification of candidate cells for biotechnological feedstocks [[21], [22], [23]]. In earlier work, we reported the use of microfluidic droplets to study microalgae at the single-cell level, including tracking the growth of individual algal cells [24], detection of ethanol-producing cyanobacteria [25], and identification of fast growing algal strains [26]. Whilst these microfluidic methods were shown to be useful for high-throughput analysis and screening of algal strains, they all used the traditional laser spot for illumination, which does not collect information across the entire microdroplet. The potential loss of fluorescence signal may hence result in reduced information about the cells.

Here we present an improved laser-induced fluorescence method combined with droplet-based microfluidic technology, which improves the analysis and screening of microalgae in a high throughput way, and which can be integrated into different workflows. A laser light sheet was created by using a Powell lens-based optical setup, which gives more reliable fluorescence detection in the microdroplet sorting system. Two distinct species of marine microalgae *Phaeodactylum tricornutum* and *Nannochloropsis gaditana* were chosen since both are genetically tractable, have sequenced genomes, and accumulate large amounts of lipids [[27], [28], [29]]. In this study, we encapsulate both species as single cells into picolitre-sized droplets. We explore whether the ability to detect the intracellular fluorescence within a discrete droplet enables the analysis of cellular processes in these species. As a proof of concept, we test whether the chlorophyll fluorescence intensity of single cells in individual microdroplets can be quantified and used to identify populations of *P. tricornutum* cells grown under different light conditions. Moreover, we investigate whether individual cells expressing green fluorescent protein (GFP) can be sorted from non-transformed cells. This is exploited to screen for transformed cells in a population, bypassing a major bottleneck in algal transformation workflows and offering an alternative way to facilitate the process of identifying genetic modified algal strains.

## 2 Materials and methods

### 2.1 Strains and growth conditions

*P. tricornutum* (CCAP 1055/1) and *N. gaditana* (CCMP 526) cultures were grown in f/2 medium under constant white fluorescent lights (60–80 μmol photons m^−2^ s^−1^) at 18 °C and 25 °C, respectively. Silica was omitted from the f/2 medium for all *N. gaditana* cultures, and for *P. tricornutum* cultures used for transformation. The growth number of the *P. tricornutum* in bulk was measured using a hemocytometer and three or more technical triplicate. The statistical analyses were performed using Origin 8.0 (OriginLab Co., USA), and the growth data of *P. tricornutum* in bulk were expressed as mean ± SD (standard deviation) of three replicates of counting.

### 2.2 Chlorophyll measurement of bulk *P. tricornutum* cultures

Cell samples (1 mL) were extracted from the bulk *P. tricornutum* cultures and centrifuged at 15000*g* for 10 min. The supernatant was discarded, and dimethylformamide (1 mL, Fisher Scientific, UK) was added to the cell pellet to extract chlorophyll pigments. The samples were agitated at room temperature for 15 min using a shaker and then centrifuged for 2 min at 10000*g*. The absorbance of the supernatant at 630 nm and 664 nm was recorded using a spectrophotometer (Helios Alpha, Thermo Scientific, UK). The total chlorophyll content of the cell samples was calculated as follows: total chlorophyll content (μg/mL) = 23.96A_630_ + 7.74A_664_ [30,31].

### 2.3 Transformation of *P. tricornutum* cells using multi-pulse square wave electroporation

*P. tricornutum* 1055/1 cells were grown in f/2 minus silica medium to an early exponential growth phase (~2–4 ∗ 10^6^ cells/mL). The cell density was determined, and the necessary resuspension volume determined to reach a final density of 2.5 ∗ 10^7^ cells per 35 μL per transformation. The cells were washed twice and resuspended in filter-sterilised 0.77 M mannitol + f/2-Si (97.5:2.5, v/v). Aliquots of 35 μL cell suspension were mixed with 5 μL of linearised plasmid (2.5 μg DNA per transformation) in a 0.2 mm gap NEPA21 electroporation cuvette. The cells were transformed using a NEPA21 multi-pulse electroporator with the following settings: Poring pulse (300 V, 5 ms length, 50 ms interval, 3–6 pulses (depending on impedance), 10% decay rate, + polarity). Transfer pulse (20 V, 50 ms length, 5 pulses, 40% decay rate, +/‐ polarity). After electroporation, the cells were transferred to 5 mL f/2 medium, incubated for 2 h in the dark at room temperature and afterwards transferred into an incubator (18 °C, constant light, no shaking) for 16–20 h. The next day, the transformed cells were transferred onto f/2 agar plates containing 75 μg/mL zeocin. The zeocin plates were placed under constant light (80 μmol photons m^−2^ s^−1^) and incubated at 18 °C for up to four weeks. Selected zeocin-resistant colonies were transferred to 200 μL fresh f/2 plus zeocin medium in 96 well plates and sub-cultured every seven days before further analysis.

### 2.4 Transformation of *N. gaditana* cells

*N. gaditana* CCMP526 was transformed using a method adapted from Radakovits et al. [29]. Cells were grown in 500 mL fresh artificial seawater (ASW) medium at 25 °C, shaking at 120 rpm and under constant light conditions (50–100 μmol photons m^−2^ s^−1^) to mid log phase. Cells were harvested by centrifugation and resuspended in 5 mL 6% ASW in 375 mM sorbitol (v/v). Aliquots of 400 μL cell suspension and 10 μg of *Sca*I-linearised plasmid were added to a 2 mm electroporation cuvette and pulsed once at 2500 V (*i.e.* 12,500 V/cm) using an Eppendorf electroporator. After electroporation, cells were recovered in 10 mL ASW under low light condition for 24 h. Afterwards, cells were pelleted by centrifugation and resuspended in 100 μL ASW before plating on ASW 1.5% agar plates containing 3 μg/mL zeocin. Colonies appeared after three to four weeks of incubation at 25 °C under constant light, at which time colonies were selected for further analysis.

### 2.5 Encapsulation and growth of *P. tricornutum* and *N. gaditana* cells in microdroplets

**SI Fig. S1:**
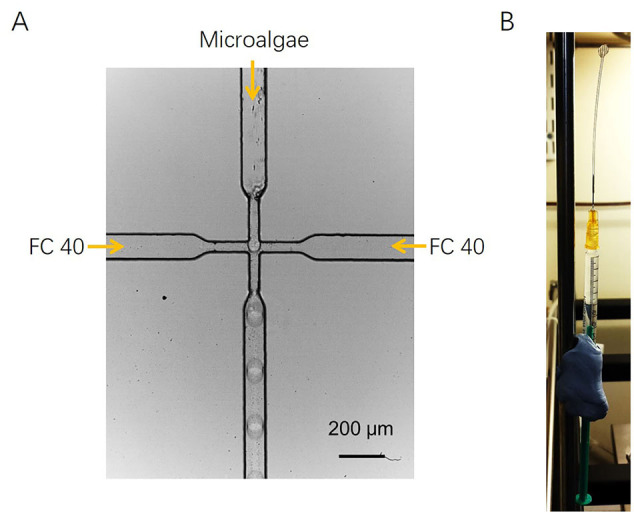
Microdroplet formation and storage. (A) Principle of microdroplet formation in microfluidic device. The diluted aqueous microalgae solution flows perpendicular to two streams of carrier oil FC 40. (B) The collected microdroplets are stored in a 1 mL syringe until further analysis.

A microfluidic device was used for encapsulation of microalgal cells into microdroplets. A suspension of cells in f/2 growth medium was injected as an aqueous phase in a flow-focusing microfluidic device of dimensions 25 μm × 50 μm (width × depth). The aqueous cell suspension flowed perpendicularly to two streams of fluorinated carrier oil Fluorinert™ FC-40 (3 M, United States) containing 2.5 wt% surfactant PicoSurf 1 (Sphere Fluidics, United Kingdom) (SI Fig. 1A). The oil streams enveloped microdroplets that budded off from the aqueous stream and flowed away from the flow-focusing junction. The size of the microdroplets was tuned by changing the flow rate of the aqueous cell suspension or fluorinated carrier oil. The microdroplets were collected and stored in a 1 mL plastic syringe over a period of ~7 days to investigate their growth (SI Fig. 1B). To monitor the growth of *P. tricornutum* and *N. gaditana* in microdroplets, microscope images were captured with an EMCCD iXonEM+ DU 897 camera (Andor Technology, United Kingdom) coupled with an IX 81 inverted microscope (Olympus, Japan). The number of cells in microdroplets was determined by counting cells using images taken in technical triplicate. The statistical analyses were performed with Origin 8.0 (OriginLab Co., USA), and the data were expressed as mean ± SD (standard deviation) of three replicates of counting.

### 2.6 Droplet-based microfluidic detection and screening of microalgae

**SI Fig. S2:**
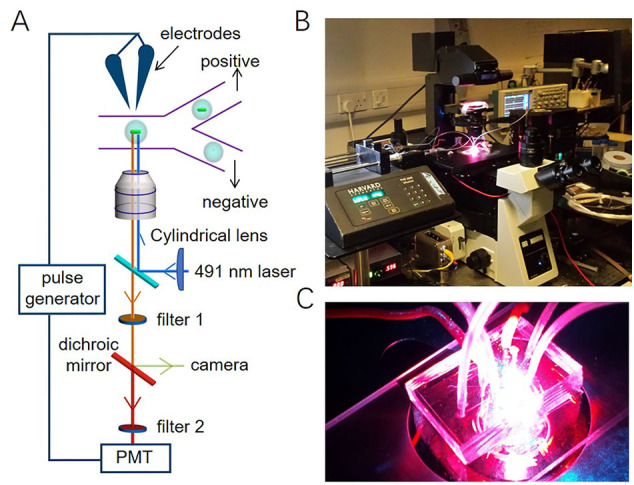
Detection and screening setup. (A) Schematic of the optical setup to detect and sort microdroplets. (B) and (C) pictures of microfluidic devices during an experiment.

To detect the fluorescence in the microdroplet, a fixed wavelength laser with excitation at 491 nm was used in the droplet-based microfluidic detection and screening setup (SI Fig. 2). The collimated laser beam passes a Powell lens and generated a laser-light sheet to go through a 20× objective (IX 73 inverted microscope, Olympus, Japan). The beam image is a long narrow slit of 491 nm illumination perpendicular to the direction of the flow in the microfluidic channel. Emitted fluorescence was filtered through a 491 nm long-pass filter to eliminate the 491 nm excitation wavelength from collected light. A dichroic filter of 633 nm was used to split light between the fast-camera (Phantom MicroEX4, Vision Research, United States) for recording the videos of the microdroplets and the photomultiplier tube (PMT) for capturing the emitted fluorescence. For chlorophyll fluorescence measurements, a 633 nm long-pass filter was mounted to the PMT so that the red fluorescence was recorded. For GFP, a 525/39 nm band-pass filter was used.

A custom written LabVIEW software on a PC with a data acquisition card (NI PCI-6251, National Instruments, United States) was used to record fluorescence intensities of cells in microdroplets. The PMT converted the emitted fluorescence into corresponding signal output voltages which were recorded by the data acquisition card. The voltage signals were used for determining whether microdroplets should be selected. When the intensity of the PMT voltage was over a defined threshold, the electrodes were activated to steer microdroplets containing the target cells to a ‘positive’ channel. Otherwise, the electrodes were off and microdroplets flowed to the ‘negative’ channel. The microdroplets flowing into the ‘positive’ and ‘negative’ channels were finally collected in a 1 mL injection syringe which was pre-filled with 100 μL f/2 medium. To release the microalgal cells, the microdroplets were demulsified by adding 100 μL 1H,1H,2H,2H-perfluorooctanol (Alfa Aesar, United Kingdom) and the microalgal cells were dispersed into f/2 medium allowing growth in bulk. All detection and sorting experiments were performed in technical replica (*n* ≥ 3). All statistical analyses were performed with Origin 8.0 (OriginLab Co., USA).

## 3 Results

### 3.1 The growth of individual *P. tricornutum* and *N. gaditana* cells in microdroplets

To monitor the growth of individual cells of *P. tricornutum* and *N. gaditana* in microdroplets, cells grown in f/2 medium were encapsulated in microdroplets with an average diameter of 50 μm with the aim of having no more than one cell per droplet. A dilute cell suspension was used to minimise the number of droplets that contain multiple cells, the number of cells in each droplet following a Poisson distribution. Conditions were established that resulted in one or none cells per droplet, leading to a droplet occupancy of around 15% (*i.e.* the majority of the droplets were empty). After encapsulation, microdroplets were stored in a 1 mL plastic syringe under continuous illumination at 80 μmol photons m^−2^ s^−1^ over seven days.

**Fig. 1:**
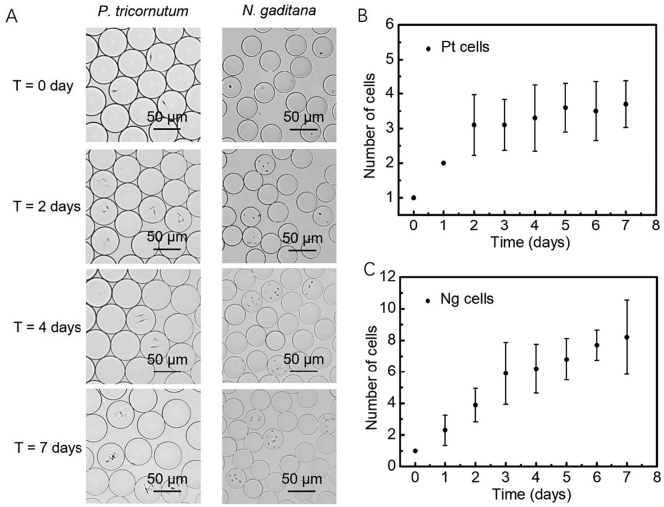
(A) Representative microscope images of the *P. tricornutum* and *N. gaditana* cells growing in microdroplets over the span of 7 days. Growth curve of (B) *P. tricornutum* cells and (C) *N. gaditana* cells in microdroplets. Starting from one cell at day zero, the number of cells per microdroplet increases over time (data reported in panels (B) and (C) as mean ± SD; *n* = 3).

**SI Fig. S3:**
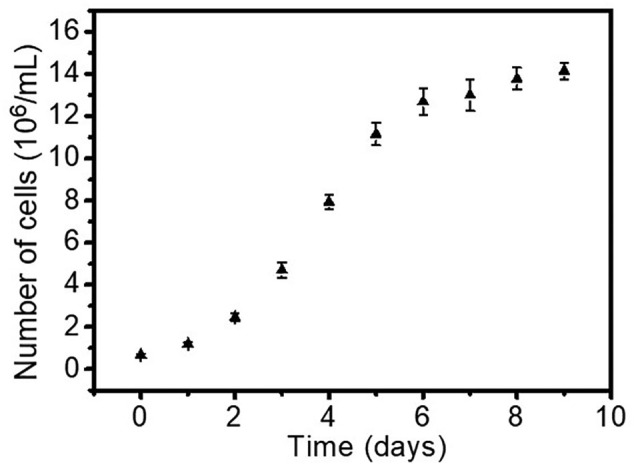
Growth curve of *P. tricornutum* cells grown in 20 mL f/2 bulk culture. (mean ± SD; *n* = 3).

Fig. 1A shows the visualization of the microdroplets over this period, with an increasing number of cells for both species indicating cell growth and division. After 7 days, the maximum number of *P. tricornutum* cells in a microdroplet was six, and for *N. gaditana* the maximum number of cells in a microdroplet was ten; there was no increase in number after this period. This equates to a cell density of 9.2 × 10^7^ cells ml^−1^ and 3.8 × 10^8^ cells ml^−1^, respectively. *P. tricornutum* cells grown in microdroplets reached a slightly higher cell density than in bulk (1.4 × 10^7^ cells ml^−1^), whilst the growth rate (Fig. 1B) was comparable with bulk conditions (SI Fig. 3). *N. gaditana* cells grown in microdroplets also have a comparable growth rate compared to bulk growth conditions, but again reached a slightly higher cell density than in bulk (9.2 × 10^7^ cells ml^−1^). The results match the observations made by Pan et al. [24] that the doubling times in microdroplets achieved for three other species (*Chlamydomonas reinhardtii*, *Chlorella vulgaris*, and *Dunaliella tertiolecta*) corresponded to those measured in bulk cultures.

**SI Fig. S4:**
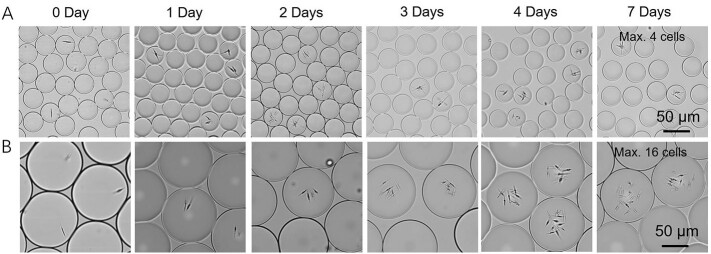
Representative microscope images of *P. tricornutum* cells growing in microdroplets of different diameter over the span of 7 days. Microdroplet diameter is 37 μm (A) or 108 μm (B).

The maximum number of algal cells in the microdroplets could be manipulated by changing the size of the droplet. In those microdroplets of ~108 μm diameter, the maximum cell number reached after 7 days was 16 *P. tricornutum* cells, whilst a ~37 μm diameter microdroplet could only accommodate four *P. tricornutum* cells (SI Fig. 4). This corresponds to a cell density of 2.4 × 10^7^ cells ml^−1^ and 1.5 × 10^8^ cells ml^−1^, respectively. Reaching different cell densities by changing the microdroplet size may be due to factors such as depletion of nutrients and cell morphology.

### 3.2 Chlorophyll fluorescence measurement of microalgae in microdroplets using an improved laser-illumination system

**Fig. 2:**
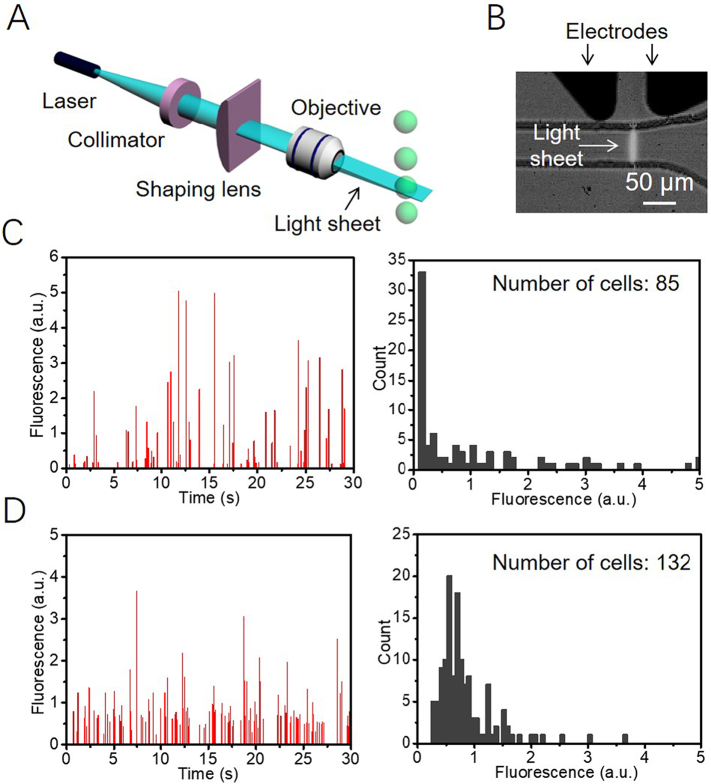
(A) Schematic diagram of the setup for generation of a laser light sheet. (B) A microscope image of the laser light sheet illumination in a microfluidic sorting channel. (C, D) Histogram of the fluorescence signals (left panel) and the corresponding fluorescence intensity distribution (right panel) from microdroplets containing wild type *P. tricornutum* cells. (C) data from microdroplets under light spot illumination; (D) data from microdroplets under light sheet illumination. The data shown in C and D is example data for a 30 second detection when run with a constant flowrate of 1800 droplets min^−1^.

**SI Fig. S5:**
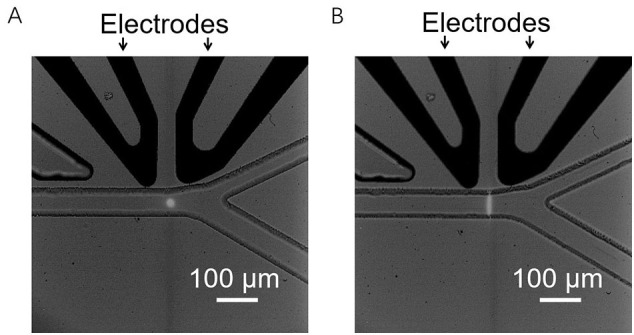
Comparison of different laser light illuminations. Image of microfluidic device with (A) laser point and (B) laser sheet configuration.

To measure the intracellular fluorescence of *P. tricornutum* and *N. gaditana* microalgae in microdroplets, the droplets were flowed through a microfluidic channel and illuminated using a 491 nm blue laser beam essentially as described previously [26]. If a microdroplet contained a cell with chlorophyll, excitation occurred, and the resulting red fluorescence was detected by a PMT. In the earlier fluorescence detection systems, the results were generated by spot illumination with a laser light (SI Fig. 5A) [25,26]. However, when the diameter of the laser spot was smaller than the microfluidic channel, the channel was not uniformly irradiated, making it difficult to get a reliable quantitative measure of fluorescence. One way to address this problem would be to use a larger size for the laser spot, but the Gaussian intensity distribution of the laser spot would cause the non-uniform illumination of the microfluidic channel [32]. We therefore decided to develop a laser sheet illumination system where the laser beam is passed through a shaping lens, producing a thin, focusable light sheet straight out of the microscope, as illustrated in Fig. 2A. Compared to our previously reported spot illumination the light sheet uniformly covers the whole microfluidic sorting channel in a perpendicular orientation to the flow direction of the microdroplets (Fig. 2B; SI Fig. 5B).

**SI Fig. S6:**
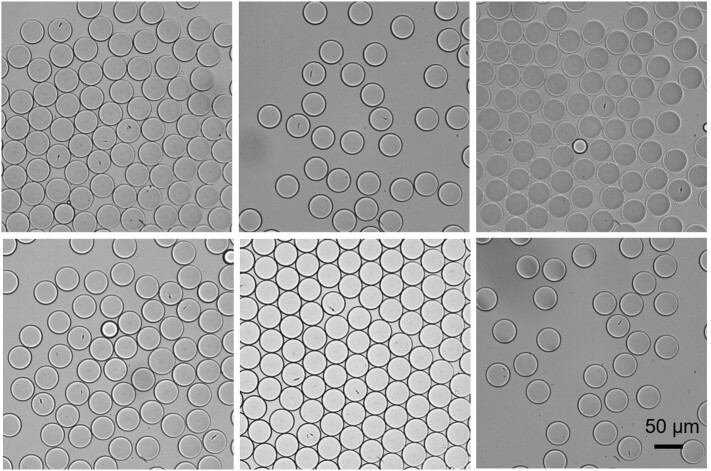
Representative microscope images of *P. tricornutum* cells encapsulated in microdroplets. The majority of droplets are empty with only around 15% occupied by *P. tricornutum* cells.

**SI Fig. S7:**
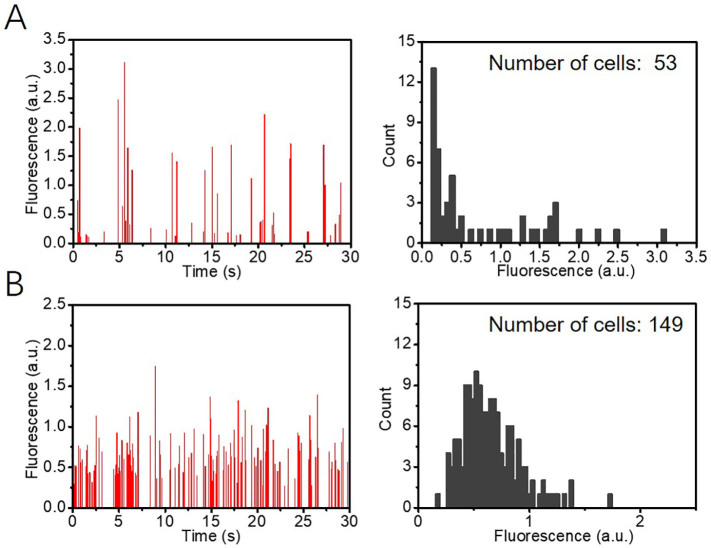
Detection of *Nannochloropsis* cells by using (A) laser spot illumination or (B) laser sheet illumination. More cells are detected when using the laser sheet configuration. The data shown in A and B is example data for a 30 second detection when run with a constant flowrate of 1800 droplets min^−1^.

Fig. 2C and D show histograms of the fluorescence signals and the corresponding fluorescence intensity distribution obtained from microdroplets containing wild type *P. tricornutum* cells when a laser light spot (Fig. 2C) or a laser light sheet (Fig. 2D) were used to irradiate the droplets. Each peak represents the chlorophyll fluorescence from the cell in one microdroplet. In a typical experiment, by counting cells from microscopy images (SI Fig. 6), we found 15% of microdroplets to contain *P. tricornutum* cells. When running with a constant flow rate of ~1800 droplets min^−1^ and considering a 15% occupancy rate, in 30 s a predicted 135 droplets would contain a cell. We were able to detect 132 peaks using laser sheet illumination (Fig. 2D). In contrast, when using a laser spot only 85 peaks were detected in the same time (Fig. 2C), indicating an efficiency of less than 65%, presumably because the size of the laser spot was too small to cover the whole microdroplet. For the smaller sized *N. gaditana* cells encapsulated in microdroplets, 149 cells were detected using laser sheet illumination, whilst only 53 cells were found in the same time under laser spot illumination (*i.e.* ~65% missed), further illustrating how the light sheet improves the detection efficiency for cells in microdroplets (SI Fig. 7). Thus, the laser light sheet resulted in a significant improvement on the reliability of the fluorescence values recorded by the PMT and will allow a wide range of applications.

### 3.3 An example for utilising the technology: identification of *P. tricornutum* cells with high chlorophyll content

**Fig. 3:**
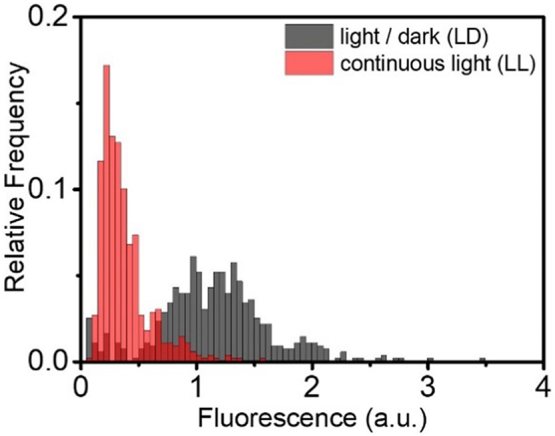
Chlorophyll fluorescence intensity histograms showing the chlorophyll fluorescence distribution for *P. tricornutum* cells cultured under a light/dark cycle of light intensity 30 μmol m^−2^ s^−1^ (black), and under continuous light of light intensity 75 μmol m^−2^ s^−1^ (red). Data is shown for a representative culture of each treatment and fluorescence was detected during independent runs using a constant flowrate of 1800 droplets min^−1^. (For interpretation of the references to colour in this figure legend, the reader is referred to the web version of this article.)

**SI Fig. S8:**
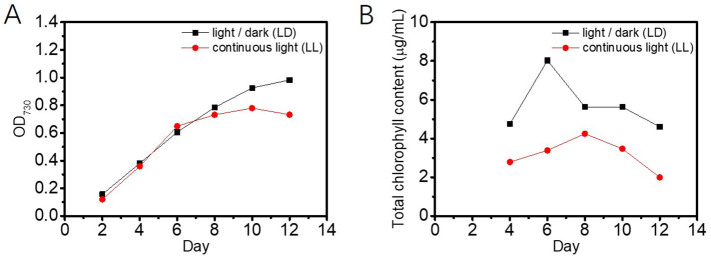
Monitoring of *P. tricornutum* cultures under different light conditions. Cultures were grown in light/dark (LD) or constant (LL) light regimes in f/2 growth medium. Every two days growth at OD730 (A) and total chlorophyll content (B) was measured under the LD (black) and LL (red) light regim0065 (data reported as mean; *n* = 2).

For *P. tricornutum* grown in bulk culture, the chlorophyll content of the cells is influenced by the light intensity and the light regime (continuous light *vs* a light/dark cycle) [33]. To test the detection capabilities of the microdroplet-based detection platform, *P. tricornutum* cells were cultured in 24-well plates under two different light regimes: a low light intensity (30 μmol m^−2^ s^−1^), light/dark regime (LD, 16 h light/8 h dark) and a continuous illumination regime (LL) of light intensity 75 μmol m^−2^ s^−1^. The growth of the cells was tracked over 12 days by measuring the OD_730_ of the cultures and the chlorophyll in the cells was extracted and measured every two days (SI Fig. 8). On day 8, cell samples were taken from each of the two cultures and were encapsulated separately into microdroplets. The chlorophyll fluorescence from the cells of each population was detected using the laser sheet platform. A comparison of the two resulting fluorescence intensity distributions showed that overall the cells cultured under the light/dark cycle had a higher chlorophyll content than the cells cultured under continuous light (mean of fluorescence distributions shifted to the right, Fig. 3). This observation agrees with our bulk measurements (SI Fig. 8) and previous experiments [33]. Additionally, since the cells in the cultures were screened individually, it was possible to observe cell-to-cell variation in the chlorophyll content, which was greater in cells cultured under light/dark conditions compared to cells grown under continuous light conditions (wider *vs* narrow distribution, Fig. 3).

### 3.4 Enrichment of the occupancy of the *P. tricornutum* and *N. gaditana* cells in microdroplets

**Fig. 4:**
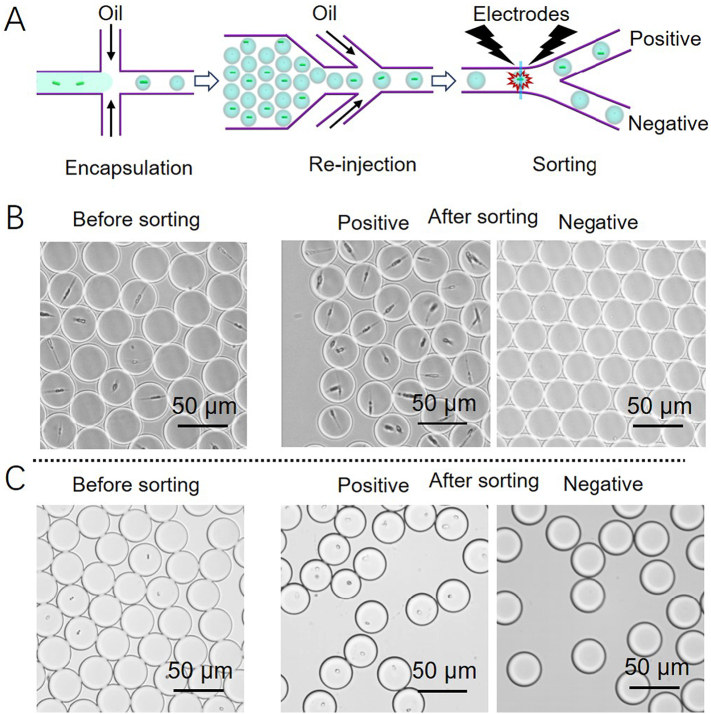
(A) Workflow of the droplet-based screening of microalgae using chlorophyll fluorescence. Representative microscope images of (B) *P. tricornutum* cells and (C) *N. gaditana* cells in microdroplets before and after sorting based on the chlorophyll fluorescence.

As explained above, to ensure no more than a single cell per droplet, the occupancy of the droplets at the beginning of the experiments is only around 15%. To enrich the proportion of droplets containing algal cells, we took advantage of the ability of the microfluidic devices to sort based on chlorophyll fluorescence. As illustrated in Fig. 4A, either *P. tricornutum* or *N. gaditana* cells were encapsulated, and samples of the microdroplets were re-injected into a sorting device, in which the chlorophyll fluorescence of the encapsulated cells was recorded and used for triggering dielectrophoresis-based droplet sorting. In brief, when chlorophyll fluorescence is detected, the PMT sends a signal to trigger the application of a waveform to the electrodes adjacent to the sorting region. An inhomogeneous electric field is then generated in the sorting device, which steers each microdroplet containing one or more cells into the upper ‘positive’ channel by dielectrophoresis, separating these occupied microdroplets from the non-fluorescent ones, which are drawn into the lower ‘negative’ channel by hydrodynamic forces (SI Video S1). To allow detection and sorting, the flow rate in a sorting device was set to ~4260 droplets min^−1^. The sorted microdroplets were collected separately from the empty microdroplets, as shown in Fig. 4B, C. The sorting process resulted in fewer than 1% of droplets in the positive channel being empty, demonstrating the accuracy of the selection of both *P. tricornutum* and *N. gaditana* cells. In the negative channel there were similarly very few false negatives. Using a microdroplet flow rate of 1 × 10^6^ droplets hour^−1^ in the sorting device, the screening rate is 1.5 × 10^5^ algal cells per hour.

### 3.5 Microdroplets screening *P. tricornutum* and *N. gaditana* cells based on GFP

**Fig. 5:**
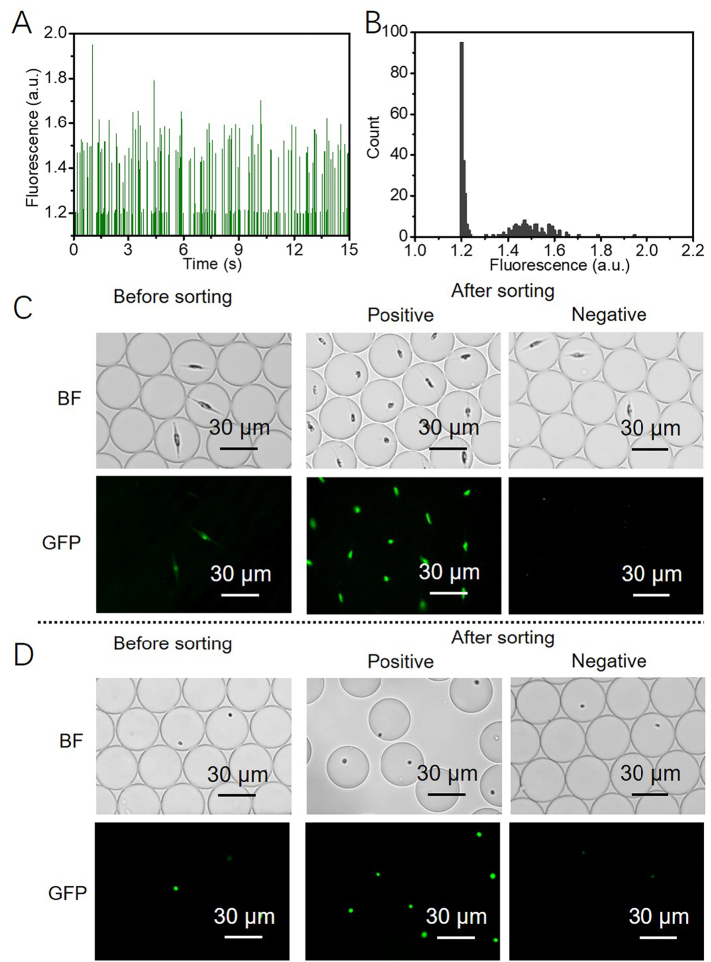
(A) Histogram of the fluorescence signals and (B) the corresponding fluorescence intensity distribution from microdroplets containing GFP-expressing and wild type *P. tricornutum* cells. The data shown in A and B is example data for a 15 s interval of detection with a flow rate of ~4500 droplets min^−1^. (C) Bright field (BF) microscope images and GFP channel of fluorescent microscope images of *P. tricornutum* cells in microdroplets before and after sorting based on GFP. Representative images are shown. (D) BF microscope images and fluorescent microscope images of *N. gaditana* cells in microdroplets before and after sorting based on GFP.

To expand the utility of the microdroplet sorting platform it is important to be able to detect and measure the fluorescence of other biotechnologically useful molecules, as well as the intrinsic chlorophyll fluorescence in microalgae. To illustrate this, a mixture of wild type and GFP expressing *P. tricornutum* cell lines was encapsulated in microdroplets, ensuring single cell occupancy, and screened based on the GFP fluorescence emitted. The results show that both GFP fluorescence and chlorophyll-based auto-fluorescence were detected by the PMT, and two different intensity distributions were observed (Fig. 5A and B). Therefore, by setting a threshold below the GFP fluorescence intensity but above auto-fluorescence, it was possible to sort GFP-expressing *P. tricornutum* cells from wild type cells. In this way, microdroplets containing GFP-expressing cells could be selected against a background of microdroplets containing either non-expressing cells or no cells (Fig. 5C). Similar results were obtained using *N. gaditana* cells (Fig. 5D). The majority of GFP-expressing cells show a narrow range of fluorescence, but the fluorescence intensity plot also demonstrates that a smaller number of cells show higher fluorescence levels (1.2 *vs* ~1.5 a. u.; Fig. 5B). The observed variation in fluorescence levels in this population could be due to position effects during the integration of the transgene [34]. Increasing the threshold for the fluorescence-based selection would allow selection for these high-expressing outliers.

### 3.6 Improving transformation workflow efficiency using microdroplet screening

Having established the methodology to sort cells based on GFP fluorescence we decided to investigate whether this approach could be used to improve the workflow for transformation of algal cells. Several protocols have been developed to introduce transgenes into the nucleus of *P. tricornutum* cells using biolistics or electroporation [35,36]. Owing to the random integration of the DNA into the nuclear genome through non-homologous recombination *via* these approaches, wide variations in transgene expression levels are seen across a transformant population as a result of both chromosome position effects and DNA rearrangements/losses during integration. This effect is also well documented in *C. reinhardtii* [34,37]. As a consequence, extensive screening is necessary to isolate suitable transformed lines that possess the unaltered transgenic DNA and stably express the transgene(s) at a desirable level. This means that transformation protocols are frequently lengthy and labour intensive.

**Fig. 6:**
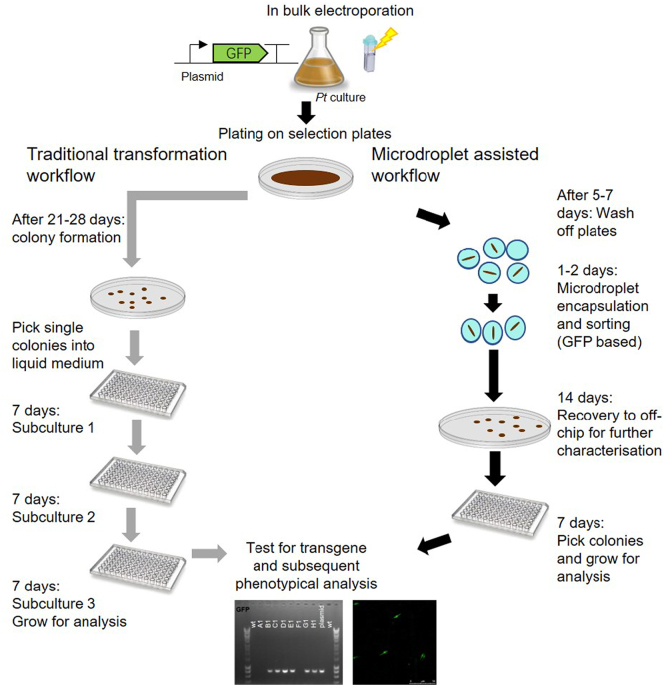
Flowchart showing the transformation workflow following the traditional route (grey arrows) or the microdroplet-assisted route (black arrows).

To speed up the identification of positive transformants, we incorporated a microdroplet step into the transformation workflow. We compared the traditional approach with this new method, transforming *P. tricornutum* cells with a plasmid carrying two independent expression cassettes (a zeocin resistance cassette and a GFP expression cassette). In our experiments, the average transformation efficiency for *P. tricornutum* is approximately 10^3^ transformed cells in 10^8^ cells. In a traditional transformation workflow, isolated *P. tricornutum* colonies, obtained three to four weeks after the transformation event, were selected and monitored for viability under selection pressure over a period of up to 21 days (three subcultures, with each lasting seven days) (Fig. 6, grey arrows). During the early subculturing around 15% of the initial selected (zeocin resistant) lines were lost, but after three subcultures the number of zeocin resistant lines remained stable. Testing for *gfp* integration and GFP expression, it was possible to detect the *gfp* gene by PCR in up to 80% of the zeocin resistant colonies. Approximately 55–60% of the obtained colonies expressed the GFP protein as determined by confocal microscopy (Table 1).

**Table 1:** Comparison between traditional and microdroplet-assisted transformation workflow. Data obtained for each workflow is based on three independent transformation events (biological replica, n = 3).

	Traditional transformation workflow	Microdroplet assisted workflow
Time between electroporation and strain confirmation	42–49 days (6–7 weeks)	20–27 days (4–4.5 weeks)
% of cells with *gfp* transgene integration	80%	98%
% of cells expressing GFP protein	57%	95%

As an alternative, the fluorescence-based microdroplet sorting platform was included within our transformation workflow (Fig. 6, black arrows). After an initial 5-7 day incubation of treated cells on selection plates, cells were washed off the plates, and approximately 1.6 × 10^6^ individual cells were encapsulated into microdroplets, so that the majority of droplets contained no more than one cell (~15% occupancy). Then, a GFP fluorescence-based sorting of these ~1.1 × 10^7^ droplets was carried out as before, collecting into the ‘positive’ channel droplets that showed a PMT signal higher than the threshold. Our experimental setup allows us to sort ~1.5 × 10^5^ algal cells per hour. After sorting and collecting droplets for 5 h, droplets from the ‘positive’ channel (~500) were re-plated on zeocin-selection plates for further off-chip analysis. Approximately 400 positive colonies were obtained. On transfer to liquid media, they were analysed for *gfp* integration by PCR and GFP protein expression by confocal microscopy. Throughout subsequent subculturing, no loss of zeocin resistant lines was observed, therefore allowing us to test for GFP expression directly during the first subculture. Compared to the traditional workflow, a higher percentage of lines tested positive for *gfp* integration (98% *vs* 80%) and for GFP expression (95% *vs* 57%) (Table 1). Thus, the microdroplet-assisted screening can help to identify transgenic clones during a transformation workflow, reducing the time for identification and confirmation of stably expressing transformants from 6–7 weeks to 4 weeks.

## 4 Discussion

We have demonstrated a successful droplet-based microfluidic method for the screening of microalgae using intracellular fluorescence, demonstrating its utility for the identification of strains with desired phenotypes. Applying the described setup, the intracellular chlorophyll and GFP fluorescence of both *P. tricornutum* and *N. gaditana* cells encapsulated in microdroplets can be analysed to discriminate between individual cells. This is a first report of the encapsulation and growth of a diatom in microfluidic devices and necessitated modifications to support viability of these cells with their unusual ‘pennate’ cell shape, and silicaceous cell walls. Diatoms are increasingly being employed in biotechnological applications given their rapid growth, ease of genetic manipulation and suitability as production platforms for high-value compounds [*e.g.* 1,6,46]. Using our method, we were able to quantify chlorophyll fluorescence intensity and use this to distinguish populations of *P. tricornutum* cells grown in different light conditions (Fig. 3), demonstrating the effect this had on single-cell growth, as well as the heterogeneity between cells. Chlorophyll fluorescence in droplet-based microfluidics has been used to monitor *C. vulgaris* growth in individual droplets under different light and nitrogen conditions [38], but in these *C. vulgaris* experiments no further sorting based on fluorescence and hence growth was undertaken.

As well as the ability to distinguish and quantify individual differences between algal cells, we were also able to separate cells from mixed populations by inclusion of a sorting step in our droplet-based microfluidic device. We first demonstrated its efficacy by sorting empty droplets from those containing algal cells, both *P. tricornutum* and *N. gaditana*, using chlorophyll fluorescence (Fig. 4), as previously shown for cyanobacteria [26]. Whilst these approaches were based on measurement of intrinsic chlorophyll fluorescence, we then extended this approach to sort *P. tricornutum* cells that had been transformed with a *gfp* construct from non-transformed or non-expressing cells to address a major bottleneck in algal transformation workflows. We demonstrated that a high throughput rate of analysis can be achieved in the microfluidic device with sorting of GFP expressing *P. tricornutum* cells from wildtype or non-expressing cells at a rate of 1.2 × 10^5^ cells h^−1^. By this means, the time taken to obtain stable highly-expressing transformants was halved compared to traditional work flows –7 weeks to 4 weeks (Table 1).

In our microdroplet-assisted transformation workflow, it was necessary to combine droplet-based sorting with a brief, five to seven days, selection step prior to encapsulation (Fig. 6). This initial selection step was necessary as the transformation rates for *P. tricornutum* are low (~1 transformed cell per 10^5^ cells), which would require running the sorting device for several hours (~1.5 × 10^5^ algal cells per hour can be sorted) to obtain sufficient numbers of transformed cells for analysis. Future technical improvements in microdroplet screening rates so that more cells could be sorted effectively might eliminate the need for any selective step before encapsulation. Similarly, improved transformation efficiencies, for example by using episome delivery *via* bacterial conjugation [39,40], would increase the proportion of transformed cells. Both these improvements would offer the means to identify transformed cell lines without any antibiotic marker. Such marker-free strains are of high interest for commercial applications. A similar droplet-based screening platform, although using the conventional laser-spot optical system, has been used for a different purpose by Kim et al. [41], in this case to identify faster growing and high lipid content *C. reinhardtii* lines from an ethyl methanesulfonate (EMS)-mutagenized population of 200,000 individuals. Eight strains showing faster growth and higher lipid content were identified by combining an initial chlorophyll fluorescence based screen with BODIPY staining and screening [41]. The design of microdroplet screening platforms, especially the droplet generation/culture module and the droplet analysis/sorting module, can vary between different platforms (reviewed in [42]) and it is difficult to compare the output of the different platforms. For our setup, we showed that for fluorescent based analysis the laser illumination across the microdroplet plays an important role and a laser sheet illumination was much more effective at cell detection within a microdroplet population in comparison to a laser spot illumination, especially when analysing small cells such as *N. gaditana* cells (2–3 μm in diameter) (Figs. 2, SI Fig. 7). We recommend using this configuration where possible.

Fluorescent proteins such as GFP or yellow fluorescent protein (YFP) have been used previously to increase the efficiency of identification of microalgal transformants. For example, in *C. reinhardtii* the target gene, encoding a patchoulol synthase, was expressed in frame with YFP. Following transformation using an antibiotic-based selection marker, *C. reinhardtii* colonies were screened for YFP fluorescence and strains with a high fluorescent signal were further cultivated and tested for patchoulol production [43]. One could envisage that patchoulol production could be further improved by using a microdroplet-based sorting approach to screen a population of these already transformed and identified lines, maybe even over several generations, for high YFP expressing cells on the single cell level. As YFP is fused to the target gene patchoulol synthase, assuming that the accumulation of the metabolite is correlated to enzyme expression, this would result in cells with higher patchoulol production.

The combination of our fluorescent-based microfluidics sorting chips with other downstream recovery, storage or printing modules will further enhance the application of the technique. Droplet based printing and collection has been used for example in microfluidic based screening and sorting of cancer cell lines [44,45]. In conclusion, microdroplet based screening platforms can improve strain identification and screening of populations on the single cell level.

The following are the supplementary data related to this article.

SI Video S1 Microfluidic-based sorting of microdroplets containing *P. tricornutum* cells. SI Video S1

## CRediT authorship contribution statement

**Ziyi Yu**: Methodology, Investigation, Data curation, Validation, Visualization, Writing - original draft; **Katrin Geisler**: Methodology, Investigation, Data curation, Validation, Visualization, Writing - original draft; **Tina Leontidou**: Investigation, Visualization, Writing - review & editing **Rosanna E. B. Young**: Resources, Writing - review & editing **Sofie E. Vonlanthen**: Resources **Saul Purton**: Funding acquisition, Writing - review & editing **Chris Abell**: Conceptualization, Funding acquisition, Supervision, Project administration, Writing - review & editing **Alison G. Smith**: Conceptualization, Funding acquisition, Supervision, Project administration, Writing - review & editing.

## Funding

This research was funded by an sLoLa grant from the UK's 10.13039/501100000268Biotechnology and Biological Sciences Research Council (BBSRC) reference BB/L002957/1 to enable an interdisciplinary approach to algal biotechnology. T. L. was funded from the associated training grant BB/M50287X/1.

## Declaration of competing interest

The authors declare that they have no known competing financial interests or personal relationships that could have appeared to influence the work reported in this paper.
